# NanI Sialidase Enhances the Action of Clostridium perfringens Enterotoxin in the Presence of Mucus

**DOI:** 10.1128/mSphere.00848-21

**Published:** 2021-12-15

**Authors:** Mauricio A. Navarro, Jihong Li, Juliann Beingesser, Bruce A. McClane, Francisco A. Uzal

**Affiliations:** a California Animal Health and Food Safety Laboratory System, School of Veterinary Medicine, University of California, Davis, San Bernardino, California, USA; b Department of Microbiology and Molecular Genetics, University of Pittsburgh School of Medicine, Pittsburgh, Pennsylvania, USA; c Instituto de Patología Animal, Facultad de Ciencias Veterinarias, Universidad Austral de Chile, Valdivia, Chile; University of Maryland Medical Center

**Keywords:** *Clostridium perfringens*, enterotoxin, mucus, NanI sialidase

## Abstract

Clostridium perfringens enterotoxin (CPE) is the main virulence factor for C. perfringens type F strains to cause human gastrointestinal diseases, which can involve lethal enterotoxemia. During type F disease, CPE encounters an adherent mucus layer overlying the intestines, so the current study evaluated if NanI potentiates CPE activity in the presence of adherent mucus. CPE alone caused more cytotoxicity transepithelial electrical resistance (TEER) and permeability to fluorescent dextran (FD) for minimal mucus-producing HT29 cells versus that in their derivative HT29-MTX-E12 cells, which produce abundant adherent mucus. However, for HT29-MTX-E12 cells, the presence of NanI significantly increased CPE binding and pore formation, which enhanced their sensitivity to CPE effects on cytotoxicity, TEER, and FD permeability. When the ability of NanI to potentiate CPE-induced enterotoxemia was then tested in a mouse small intestinal loop enterotoxemia model, a pathophysiologically relevant 50 μg/mL dose of CPE did not kill mice. However, the copresence of purified NanI resulted in significant CPE-induced lethality. More CPE was detected in the sera of mice challenged with 50 μg/mL of CPE when NanI was copresent during challenge. The copresence of NanI and CPE during challenge also significantly increased intestinal histologic damage compared to that after challenge with CPE alone, suggesting that NanI enhancement of CPE-induced intestinal damage may increase CPE absorption into blood. Overall, these results indicate that (i) mucus inhibits CPE action and (ii) NanI can potentiate CPE action in the presence of mucus, which may help explain why type F strains that produce relatively low levels of CPE are still pathogenic.

**IMPORTANCE** NanI is a sialidase produced by some Clostridium perfringens type F strains. Here, we found that NanI can significantly increase the action of C. perfringens enterotoxin (CPE), which is the main toxin responsible for severe human enteric disease caused by type F strains. This effect likely helps to explain why even some type F strains that produce small amounts of CPE are pathogenic.

## INTRODUCTION

Clostridium perfringens causes disease in both humans and other animals (1, 2). The variety of diseases caused by this pathogen is mainly mediated by its production of ∼20 different exotoxins (2, 3). Considerable variation in toxin production patterns exists among C. perfringens strains, which allows classification of these isolates into seven types (A to G) based on the presence of genes encoding six main toxins, alpha-toxin (CPA), beta-toxin (CPB), epsilon-toxin (ETX), iota-toxin (ITX), necrotic beta-like-toxin (NetB), and enterotoxin (CPE) (4).

C. perfringens type F strains encode both CPA and CPE (4). This genotype is associated with a very common acute human foodborne disease referred to as C. perfringens type F food poisoning (5). Type F strains are also responsible for chronic gastrointestinal (GI) diseases, including ∼5 to 10% of all cases of nonfoodborne GI diseases such as antibiotic-associated diarrhea (AAD) and sporadic diarrhea (6, 7). In people with severe constipation or fecal impaction, type F infections can be lethal; this severity apparently involves enterotoxemia characterized by absorption of CPE from the intestine into the circulation, where this toxin then affects nonintestinal organs (8–10). Studies using a mouse small intestinal loop challenge model reinforced this hypothesis by demonstrating that, when CPE is absorbed from the intestines, it causes a lethal enterotoxemia by inducing hyperpotassemia that is presumed to cause cardiac arrest (11).

During type F disease, CPE is produced and released when C. perfringens sporulates in the intestines (5, 12). CPE is a 35-kDa protein that belongs structurally to the aerolysin pore-forming toxin family (13, 14). CPE receptors include several claudins, which are key components of tight junctions located between enterocytes (15). CPE kills host cells when it oligomerizes to form a small pore in plasma membranes (16). High CPE concentrations cause the formation of many CPE pores, which allows a massive Ca^+2^ influx to trigger necroptosis, while low CPE doses induce a smaller Ca^+2^ influx through fewer CPE pores to cause classical caspase-3-mediated apoptosis (17–19).

Sialidases are enzymes that release sialic acids from complex glycoconjugates present on host cell surfaces and in mucus found in the respiratory and gastrointestinal tracts (20, 21). C. perfringens produces up to three sialidases, including the cytoplasmic NanH sialidase and the exosialidases NanJ and NanI (22). For strains that produce NanI, this enzyme usually accounts for the preponderance of their exosialidase activity during vegetative growth (22–26).

Several *in vitro* and *in vivo* studies by our research group have suggested that NanI sialidase could be an important factor involved in C. perfringens-mediated intestinal diseases. For instance, this sialidase facilitates the adherence of NanI-producing type C, D, and F strains to enterocyte-like Caco-2 cells by modifying the surface of those host cells (23, 24). Similar NanI-induced enhancement of C. perfringens adherence likely helps to explain the increased colonization of the mouse intestines by type F strain F4969, where lower numbers of an F4969 *nanI* mutant than of wild-type F4969 were recovered from all intestinal segments after 4 days of incubation (27). NanI sialidase can also be an important growth and survival factor for C. perfringens in the presence of Caco-2 cells or semipurified mucin (28).

NanI sialidase has also been shown to increase CPE-induced cytotoxic effects on Caco-2 cells (29), as well as ETX-induced cytotoxic effects in MDCK cells (23) and CPB-induced cytotoxic effects in HUEVC cells (29). While demonstrating the ability of NanI to enhance the cytotoxic effects of CPE on Caco-2 cells is informative, Caco-2 cells produce minimal mucus (30). This differs significantly from the *in vivo* situation during type F disease, in which the intestines are covered by an adherent mucus layer (31). Since mucus is rich in sialylated proteins like mucins, we hypothesize that NanI enhancement of CPE activity may be even stronger in the presence of adherent mucus. The current study first tested that hypothesis *in vitro* and then directly explored whether NanI can enhance CPE-induced enterotoxemia in a mouse small intestinal loop challenge model.

## RESULTS

### Measurement of sialidase activity in mouse small intestinal loops infected with C. perfringens type F strain F4969 or its *nanI* null mutant.

For subsequent *in vitro* and *in vivo* experiments using purified NanI, it was important to employ physiologically relevant levels of NanI activity that are present during intestinal infection by NanI-producing type F strains. Therefore, this study first determined how much NanI activity was present in the intestines after a 10-h infection with F4969. This strain was chosen because it is a representative type F human nonfoodborne disease strain that induces *in vitro* sialidase activity (mainly attributable to NanI) typical of NanI-positive type F strains (24). A 10-h infection was chosen to assess NanI production in the intestines because this time point falls within the 6- to 24-h incubation time for C. perfringens type F food poisoning, according to the Centers for Disease Control and Prevention (https://www.cdc.gov/foodsafety/diseases/clostridium-perfringens.html).

**FIG 1:**
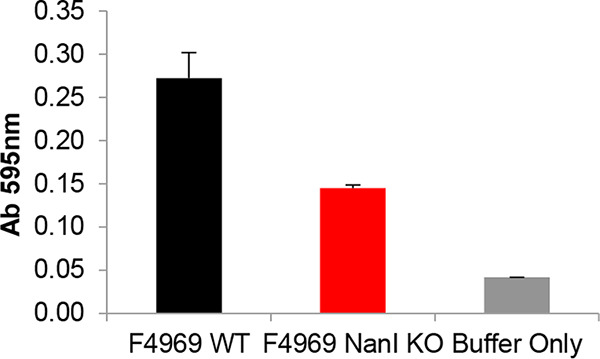
Production of sialidase activity by C. perfringens F4969, an F4969 *nanI* null mutant, or Hanks balanced salt solution (HBSS) buffer in the mouse small intestine. Sialidase activity present in supernatants of small intestinal contents from mouse intestinal loops at 10 h postinfection with ∼10^8^ CFU of wild-type F4969 (F4969 WT), a *nanI* null mutant (F4969 *nanI* knockout [KO]), or HBSS buffer alone. All data show the mean values using 6 mice. Error bars indicate standard deviation (SD).

As shown in Fig. 1, 10-h sialidase activity levels (absorbance at 595 nm [Ab_595_]) were significantly higher in supernatants of the small intestinal content collected from mice challenged with the wild-type strain than in those receiving the *nanI* knockout (KO) mutant strain or Hank’s balanced salt solution (HBSS) alone. This difference in sialidase activity between the wild-type parent and the *nanI* null mutant, which was ∼0.13, is attributable to NanI activity. The remaining differences in sialidase activity measured between loops challenged with the *nanI* null mutant versus HBSS alone are due to the production of other sialidases by F4969, which also has the genes encoding both NanH and NanJ sialidases (24).

Based upon these *in vivo* infection results, sialidase activity equivalent to ∼0.13 was used to perform all subsequent *in vitro* and *in vivo* experiments involving purified NanI.

### Comparison of sialic acid release from HT-29 (HT29) or HT29-MTX-E12 (MTX-E12) cells after NanI treatment.

**FIG 2:**
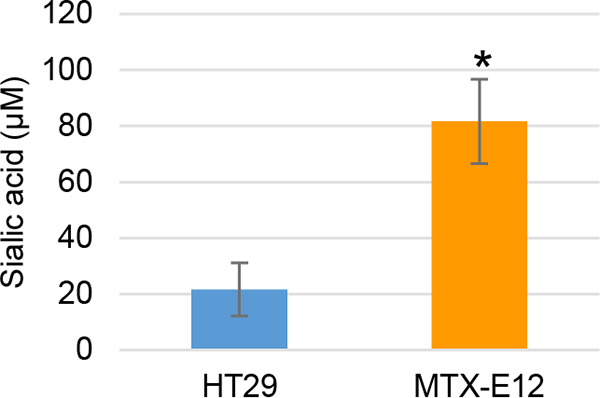
Sialic acid generation from the HT29 or MTX-E12 cell lines using the *in vivo* concentration of NanI sialidase activity determined in Fig. 1. Purified NanI (at activity levels equivalent to the NanI sialidase activity determined in Fig. 1) dissolved in HBSS was applied to HT29 or MTX-E12 cell lines for 1 h at 37°C. Sialic acid was then measured in the supernatant of each cell line. Shown is the mean of three repetitions. Error bars indicate SD. *, *P* < 0.05 relative to culture with HT29 cells.

Our previous study (29) showed that NanI can enhance the binding and cytotoxic activity of CPE on Caco-2 cells. While Caco-2 cells produce minimal amounts of mucus, adherent mucus overlays the intestines. Since mucus is heavily sialylated (32), we hypothesized that NanI may be even more impactful for CPE binding and activity in the presence of substantial levels of adherent mucus. To test this hypothesis, the current study used the MTX-E12 cell line to evaluate NanI contributions to CPE action in the presence of adherent mucus. MTX-E12 cells are derived from the minimally mucus-producing human enterocyte-like cell line HT-29 (HT29) (33) but, as confirmed by our previous study (34), HT29-MTX-E12 (MTX-E12) cells make much more adherent mucus than do HT29 cells. Therefore, the current study determined whether NanI is active on adherent mucus by comparing how much sialic acid is released from HT29 or MTX-E12 cells by purified NanI used at activity levels equivalent to those produced *in vivo* during F4969 intestinal infection (Fig. 1). Results of this experiment showed that, compared to HT29 cells, NanI generated significantly more sialic acid release from MTX-E12 cells (Fig. 2).

### NanI effects on CPE-induced cytotoxicity for HT29 cells and MTX-E12 cells.

**FIG 3:**
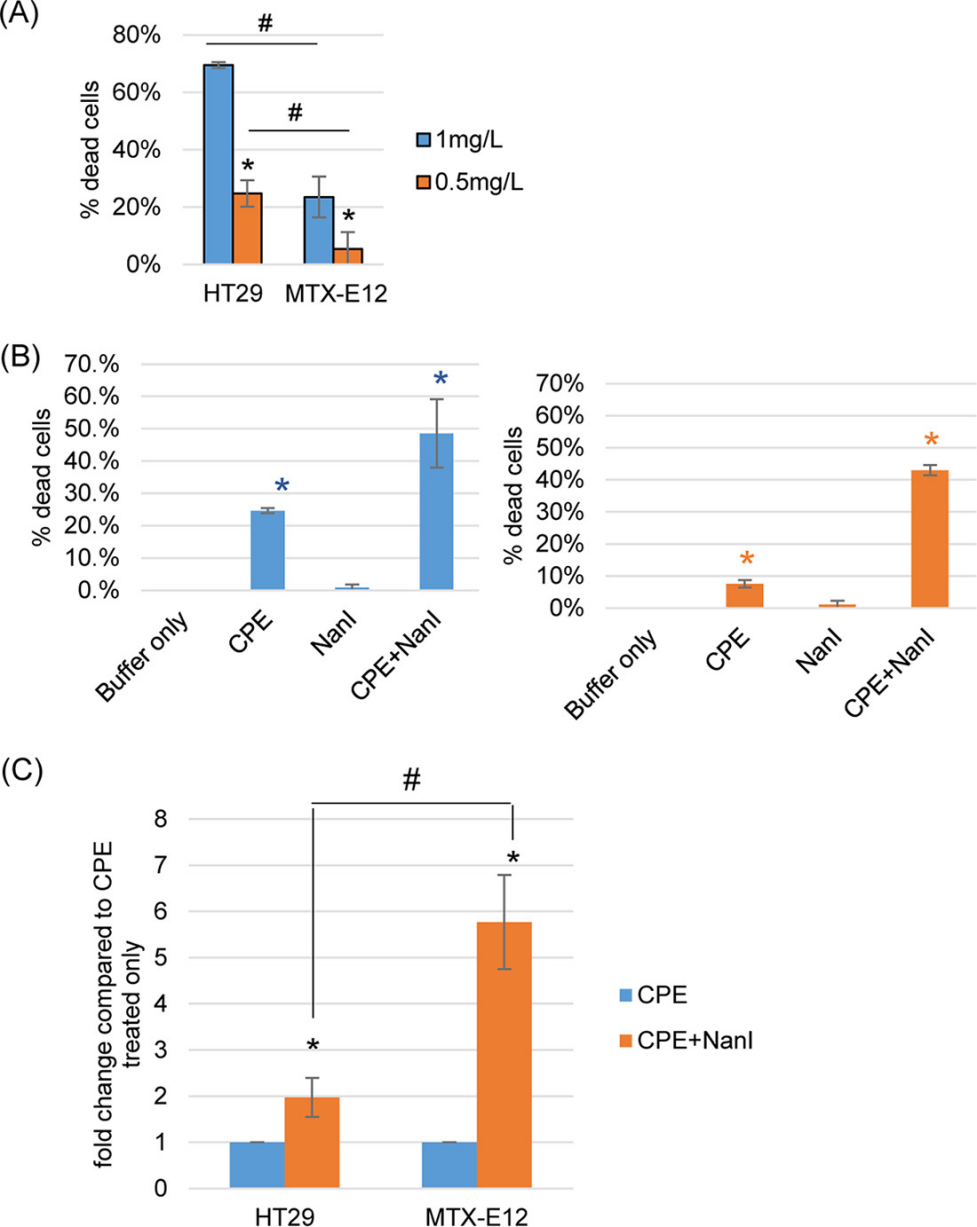
Comparison of CPE-induced cytotoxicity for HT29 and MTX-E12 cells. (A) Percentage of dead HT29 or MTX-E12 cells in cultures treated at 37°C for 1 h with HBSS containing 0.5 mg/L or 1 mg/L CPE. Shown are the mean values from three independent experiments; error bars indicate SD. *, *P* < 0.05 relative to the effects of treatment with 0.5 mg/L CPE. #, *P* < 0.05 relative to HT29 cells. (B) Percentage of dead cells in HT29 (left) or MTX-E12 (right) cultures treated at 37°C for 1 h with HBSS containing 0.5 mg/L CPE in the presence or absence of NanI. Shown are the mean values from three independent experiments; error bars indicate SD. *, *P* < 0.05 relative to buffer only. (C) Relative NanI-induced increase in CPE cytotoxicity for HT29 or MTX-E12 cells. The value of each bar indicates the calculated fold change after treatment with NanI and CPE relative to CPE treatment alone. Shown are the mean values from three independent experiments; error bars indicate SD. *, *P* < 0.05 relative to CPE treatment only; #, *P* < 0.05 relative to HT29 cells.

To evaluate whether HT29 cells and MTX-E12 cells are sensitive to CPE, those two cell lines were first treated for 1 h with 0.5 or 1 μg/mL CPE in the absence of NanI. When cytotoxicity was then evaluated, the results (Fig. 3A) showed that both cell lines are sensitive to CPE in a dose-dependent manner. However, equivalent CPE doses induced significantly higher cytotoxicity for HT29 cells than for MTX-E12 cells.

Since the 1 μg/mL concentration of CPE already caused >70% cytotoxicity to HT29 cells and we hypothesized that the presence of NanI would further increase CPE activity, the lower CPE concentration (0.5 μg/mL) was chosen for use in further experiments to test our hypothesis that NanI can be particularly impactful for increasing CPE cytotoxicity in the presence of mucus. After treatment for 1 h with HBSS containing 0.5 μg/mL CPE in the presence of NanI activity levels equivalent to the NanI levels present during intestinal infection of mice by F4969 (as deduced in Fig. 1), both cell lines exhibited significantly more cytotoxicity compared to their treatment with HBSS containing the same CPE dose alone (no NanI), HBSS containing the same NanI concentration (no CPE), or HBSS alone (Fig. 3B). Furthermore, the relative difference in cytotoxicity caused by CPE in the presence of NanI was significantly greater for the strongly mucus-producing MTX-E12 cell line than for the HT29 cell line (Fig. 3C). These results indicated that NanI has a proportionately greater potentiating effect on CPE-induced cytotoxicity in cells producing adherent mucus.

### The presence of NanI increases CPE binding and CH-1 complex formation proportionately more in MTX-E12 than in HT29 cells.

**FIG 4:**
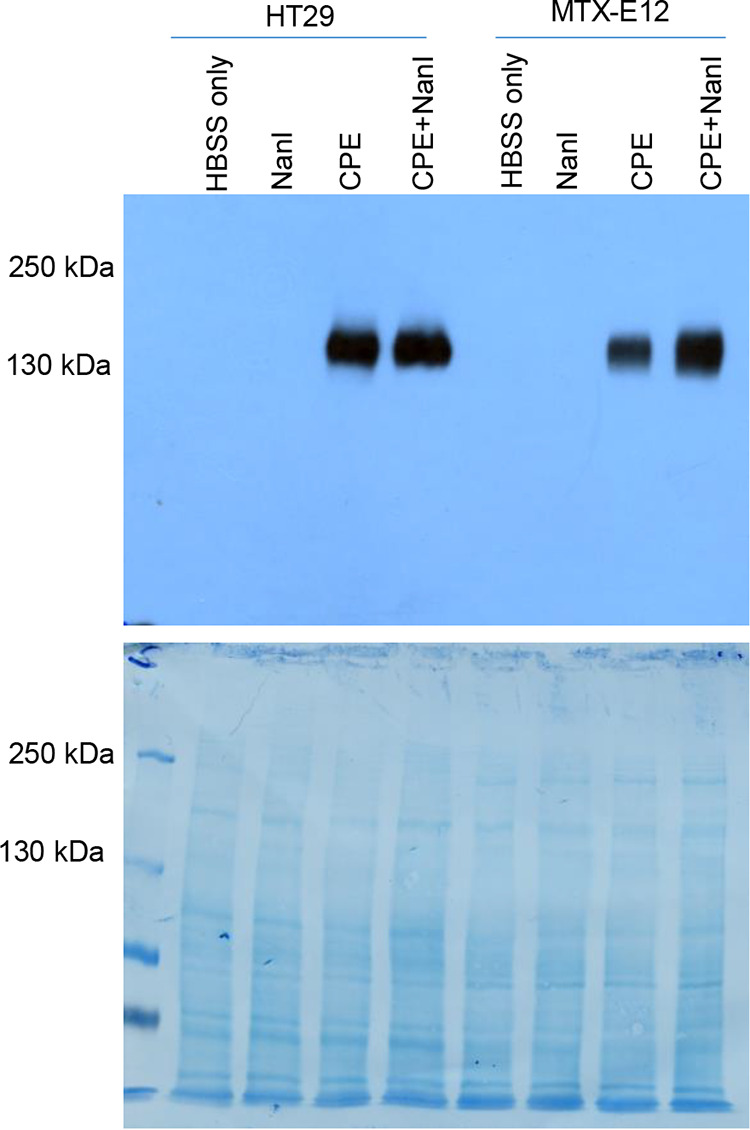
CPE large complex formation in HT29 and MTX-E12 cells. Three-week cultures of HT29 or MTX-E12 cells were treated with HBSS buffer or HBSS containing NanI, CPE, or CPE and NanI for 1 h at 37°C. Following this incubation, the cells were collected and lysed in radioimmunoprecipitation assay (RIPA) buffer with protease inhibitor and Benzonase nuclease. Total proteins were then separated by electrophoresis using 6% polyacrylamide gels containing SDS, and CPE Western blotting was performed (upper). This blot displays a representative result of three experimental repetitions. To ensure equal levels of proteins were loaded for all samples, the same polyvinylidene difluoride (PVDF) membrane was stained with Coomassie blue (lower) after CPE Western blotting.

Formation of the CH-1 CPE-containing pore complex, which is a consequence of CPE binding and oligomerization (35), is required for CPE to cause cytotoxicity in host cells (36). Therefore, formation of the CH-1 pore complex was assessed (Fig. 4) in both HT29 and MTX-E12 cells treated with (i) HBSS only (negative control), (ii) HBSS containing NanI at equivalent NanI activity to that produced by F4969 in the intestine, (iii) HBSS containing 0.5 μg/mL CPE, or (iv) HBSS containing those same concentrations of CPE and NanI together.

Results of those studies showed that, as expected, the CH-1 pore complex was absent from cells treated with HBSS or with HBSS plus NanI. When the treatment was HBSS containing CPE (no NanI), CH-1 complex was detected in both HT29 cells and MTX-E12 cells. In the absence of NanI, treatment with the same CPE concentration dissolved in HBSS caused formation of more CH-1 complex in HT29 cells than that in MTX-E12 cells, offering an explanation for why this CPE dose caused more cytotoxicity in HT29 cells versus MTX-E12 cells in the experiment shown in Fig. 3. Notably, the presence of NanI during CPE treatment increased CH-1 complex formation levels proportionately more for MTX-E12 cells compared to those of similarly cotreated HT29 cells (Fig. 4). PVDF membrane staining confirmed equal loading of all lanes.

These CPE Western blot results also indicated that the larger amount of CH-1 complex formed by cells in the presence of NanI is due to increased CPE binding.

### Effects of NanI on the barrier and permeability properties of HT29 and MTX-E12 cell monolayers.

CPE can affect intestinal permeability, leading to its absorption into the circulation to cause lethal host enterotoxemia (11). Therefore, we characterized NanI effects on the barrier and permeability properties of highly confluent monolayers of HT29 cells and MTX-E12 cells in the presence or absence of 0.5 μg/mL CPE.

First, the electrical resistance properties of polarized monolayers in 3-week Transwell cultures of HT29 or MTX-E12 cells were compared after treatment with HBSS containing NanI at activity levels equivalent to those present in the intestines of F4969-infected mice. For comparison, the effects of HBSS buffer alone or HBSS containing 0.5 μg/mL CPE (no NanI) were also assessed. Last, the effects of combined treatment with these levels of both CPE and NanI were evaluated.

**FIG 5:**
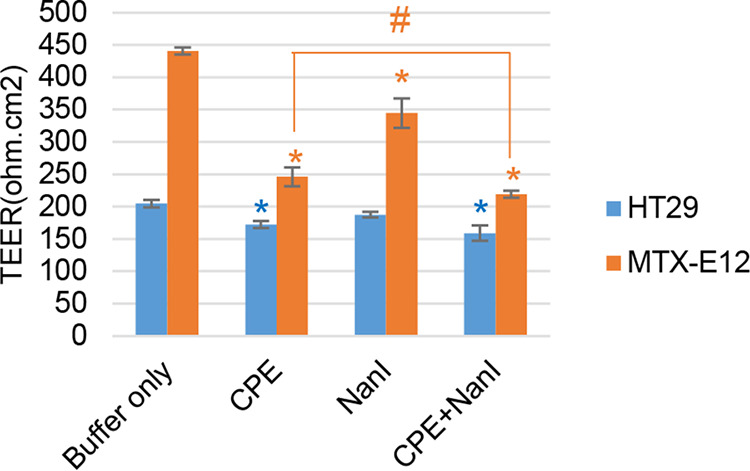
Comparison of NanI and CPE effects on transepithelial electrical resistance (TEER) of HT29 and MTX-E12 cells. HT29 or MTX-E12 cells were seeded into 12-well Transwell plates. After 21 days of culture, TEER was measured after treatment with HBSS buffer alone or treatment with HBSS plus NanI only, CPE only, or the same concentrations of NanI and CPE. All treatments were performed at 37°C for 1 h. Shown are the mean values from three independent experiments; error bars indicate SD. Blue asterisk (*), *P* < 0.05 relative to buffer only for HT29 cells; orange asterisk (*), *P* < 0.05 relative to buffer only for MTX-E12 cells; #, *P* < 0.05 relative to CPE only for MTX-E12 cells.

Results indicated that, when incubated in buffer alone, transepithelial electrical resistance (TEER) was much higher in MTX-E12 than in HT29 cells (Fig. 5). Treatment with 0.5 μg/mL CPE alone (no NanI) caused a significant drop in TEER for both cell lines during the experiment. NanI treatment alone caused a drop in TEER for both HT29 and MTX-E12 cells, although this effect only reached statistical significance for the MTX-E12 cells. It is of note that the culture TEER values for both cell lines showed even larger TEER decreases when treated with both CPE and NanI (Fig. 5). Moreover, when treated with both NanI and CPE, cultures of MTX-E12 cells showed a proportionately greater drop in TEER compared to the decrease in TEER detected for similarly treated cultures of HT29 cells (Fig. 5).

**FIG 6:**
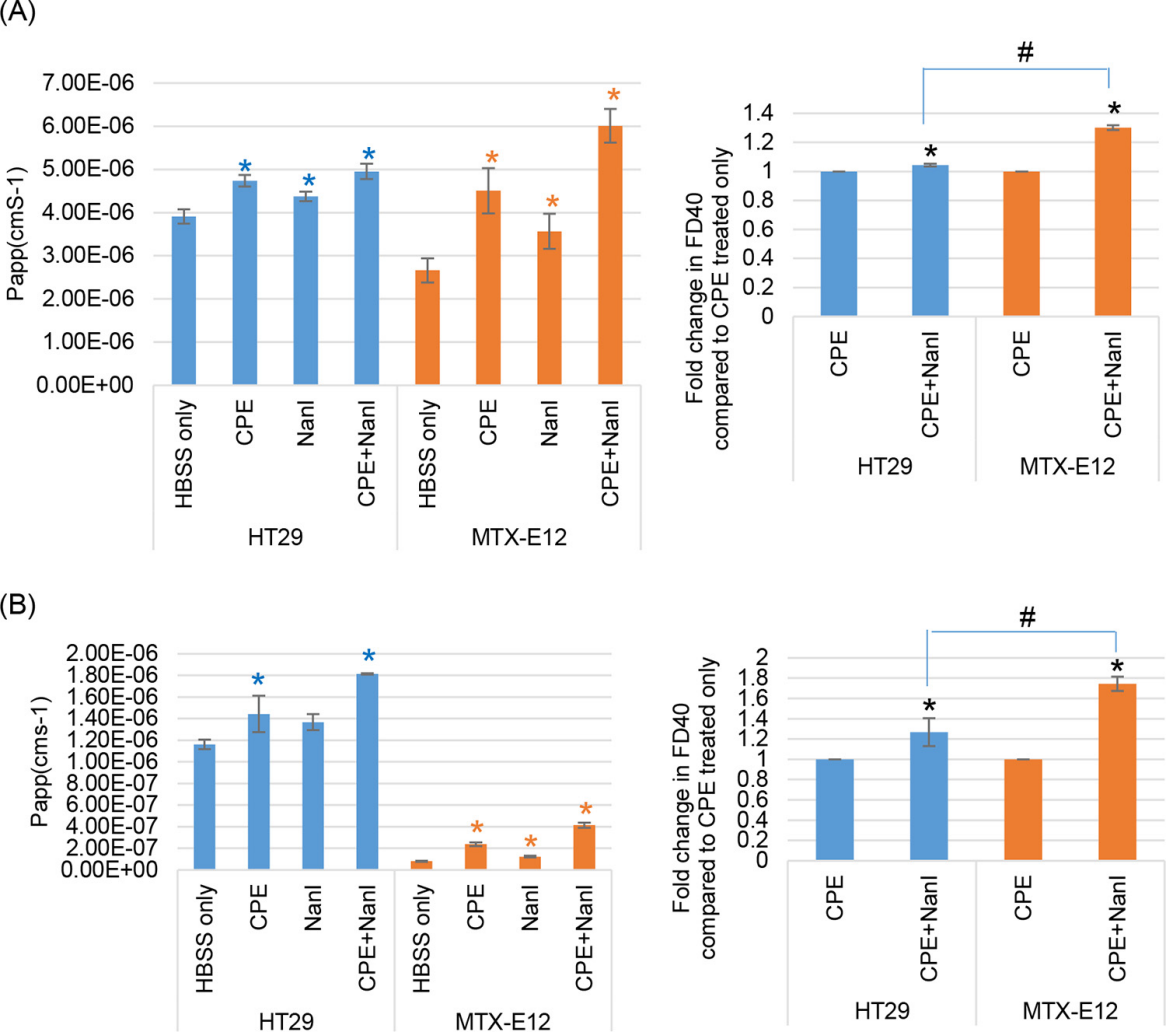
Effects of NanI and CPE, alone or together, on paracellular permeability properties of HT29 and MTX-E12 cells. Paracellular flux of fluorescein isothiocyanate (FITC)-labeled 4-kDa dextran (FD4) (A) or FITC-labeled 40-kDa dextran (FD40) (B) across monolayers of HT29 or MTX-E12 cells. After 21 days of growth, Transwell cultures of confluent, polarized HT29 or MTX-E12 cells were treated with HBSS only or with HBSS containing CPE, NanI, or the same amounts of CPE and NanI; these treatment buffers also contained FD4 or FD40 (at a final concertation of 1 mg/mL). After incubation for 1 h at 37°C, the amount of FD4 or FD40 present in the bottom chamber of the Transwell plate was measured using a BioTek Synergy fluorescence multiplate reader. Shown are the mean values from three independent experimental repetitions; error bars indicate SD. Blue asterisk (*), *P* < 0.05 relative to buffer only for HT29 cells; orange asterisk (*), *P* < 0.05 relative to buffer only for MTX-E12 cells. #, *P* < 0.05 for MTX-E12 cells relative to HT29 cells after treatment with CPE + NanI.

To evaluate NanI effects on macromolecule permeability, the movement of 4-kDa or 40-kDa fluorescein isothiocyanate (FITC)-labeled dextrans across confluent Transwell culture monolayers of HT29 and MTX-E12 cells was assessed after treatment with HBSS containing NanI. For comparison, the effects of HBSS alone or HBSS containing 0.5 μg/mL CPE (but no NanI) were also assessed. The results (Fig. 6A and B, left) indicated that, in the presence of HBSS alone, both cell lines were more permeable to 4-kDa FITC-labeled dextran (FD4) than to 40-kDa FITC-labeled dextran (FD40), likely due to the much smaller size of FD4 compared to that of FD40. However, when incubated in HBSS, there was significantly less permeability of FD4 or, particularly, FD40 in MTX-E12 cell cultures compared to that in HT29 cell cultures, supporting an impact of adherent mucus on permeability properties.

Treatment with HBSS containing CPE alone (no NanI) caused significant increases in FD4 and FD40 permeability in both cell lines. The presence of NanI in HBSS also significantly increased the permeability of both cell lines to FD4 and the permeability of MTX-E12 cells, but not that of HT29 cells, to FD40. However, when those same concentrations of NanI and CPE were copresent in HBSS during treatment, there was a significant increase in FD4 and FD40 permeability for both cell lines compared to treatment with HBSS containing CPE or NanI alone. This NanI enhancement of FD permeability was proportionately greater for the MTX-E12 cells compared to that in HT29 cells (Fig. 5A and B, right), consistent with the TEER results.

### Reduction of mucus affects MTX-E12 cell TEER, CPE cytotoxicity, and CH-1 complex formation.

To evaluate specifically whether the observed differences between HT29 and MTX-E12 cells with respect to background TEER and CPE effects on cytotoxicity or TEER involved the substantial production of adherent mucus by MTX-E12 cells, MTX-E12 cells were pretreated with the mucolytic agent *N*-acetyl cysteine (NAC). As expected from the literature (34, 37), NAC pretreatment of MTX-E12 cells reduced adherent levels of Muc5Ac relative to those in untreated MTX-E12 cells, according to Muc5Ac Western blot analyses (see Fig. S1A in the supplemental material). Supporting the presence of mucus affecting their TEER properties, these NAC-pretreated cells showed lower TEER compared to that of untreated MTX-E12 cells when cultured in the absence of CPE or NanI (Fig. S1B).

10.1128/mSphere.00848-21.1 FIG S1 Effects of *N*-acetyl cysteine (NAC) treatment on the Clostridium perfringens enterotoxin (CPE) sensitivity of MTX-E12 cells. (A) Muc5Ac Western blot analysis for MTX-E12 cells or MTX-E12 cells treated with NAC (mucus depleted) (left). This blot displays a representative result for three experimental repetitions. To ensure that equal levels of proteins were loaded for all samples, the same polyvinylidene difluoride (PVDF) membrane was stained with Coomassie blue after Muc5Ac Western blotting (right). The size of proteins in kDa is shown at left. (B) Comparative effects of NanI and CPE on transepithelial electrical resistance (TEER) of MTX-E12 cells or NAC-pretreated (mucus-depleted) cells. TEER was measured after treatment with Hanks balanced salt solution (HBSS) buffer alone or with HBSS containing NanI only, CPE only, or NanI and CPE. Shown are the mean values from three independent experimental repetitions. Error bars indicate the standard deviation (SD). *, *P* < 0.05 relative to MTX-E12 cells. (C) Comparison of NanI effects on the cytotoxicity of 0.5 μg/mL CPE for MTX-E12 cells versus NAC-pretreated (mucus-depleted) cells. The value of each bar indicates the calculated fold change between treatments where NanI was added with CPE relative to CPE treatment only. Shown are the mean values from three independent experimental repetitions. Error bars indicate SD. *, *P* < 0.05 relative to CPE treatment only. (D) Comparison of CPE large complex formation in MTX-E12 cells versus NAC-pretreated (mucus-depleted) cells. MTX-E12 cells or mucus-depleted cells were treated with HBSS buffer or with HBSS buffer containing NanI only, CPE only, or CPE and NanI for 1 h at 37°C. Following incubation, cells were collected and lysed in radioimmunoprecipitation assay (RIPA) buffer with protease inhibitor and Benzonase nuclease. Supernatants of those samples were then separated on 6% polyacrylamide gels containing SDS, and CPE Western blotting was then performed (upper). This blot displays a representative result of three experimental repetitions. To ensure that equal levels of proteins were loaded for all samples, the same PVDF membrane was stained with Coomassie blue after CPE Western blotting (lower). The minor band above CH-1 in MTX-E12 cells treated with both NanI and CPE is likely a second CPE complex named CH-2 (46). Formation of this complex was also visible in other samples if the blot was exposed longer. Download FIG S1, TIF file, 3.2 MB. Copyright © 2021 Navarro et al. 2021 Navarro et al. https://creativecommons.org/licenses/by/4.0/ This content is distributed under the terms of the Creative Commons Attribution 4.0 International license.

Consistent with the presence of mucus reducing CPE sensitivity and NanI degrading that mucus to increase CPE sensitivity, NAC-depleted cells treated with either CPE or NanI showed a significant decrease in their TEER compared to that of similarly treated MTX-E12 cells (Fig. S1B). TEER was further reduced in NAC-depleted cells versus MTX-E12 cells when these cells were treated simultaneously with both CPE and NanI, although this effect did not reach statistical significance.

Also supporting the involvement of mucus degradation in NanI enhancement of CPE activity, the NAC-pretreated cells showed proportionately less cytotoxicity than MTX-E12 cells after combined treatment with CPE and NanI (Fig. S1C). CH-1 complex formation matched these cytotoxicity results (Fig. S1D), with NanI causing less enhancement of CPE complex formation in NAC-depleted cells compared to that in MTX-E12 cells. Collectively, these results support the presence of mucus protecting against CPE activity and NanI enhancement of CPE activity involving the effects of this sialidase on mucus.

### Influence of NanI sialidase on CPE-induced enterotoxemia in mice.

Since (i) NanI enhances CPE TEER and permeability effects in the presence of adherent mucus (Fig. 5 and 6), (ii) the intestines are covered by adherent mucus (31, 38), and (iii) CPE is believed to cause lethal enterotoxemia in mice and humans when it is absorbed from the intestines into the circulation (11), we postulated that the presence of NanI can impact CPE-associated enterotoxemia in a mouse model (11, 39). This was tested by challenging mice via intraintestinal loop inoculation with HBSS alone, HBSS with purified CPE (50 μg/mL), HBSS with purified NanI sialidase (equivalent to the amount of NanI activity produced by F4969 *in vivo*), or HBSS with those same concentrations of both CPE and NanI. A 50 μg/mL CPE dose was chosen for this initial challenge experiment since this concentration is pathophysiologically relevant (40) and, by itself (no NanI present), has been shown previously (11) to induce little or no enterotoxemic lethality in this mouse assay.

**FIG 7:**
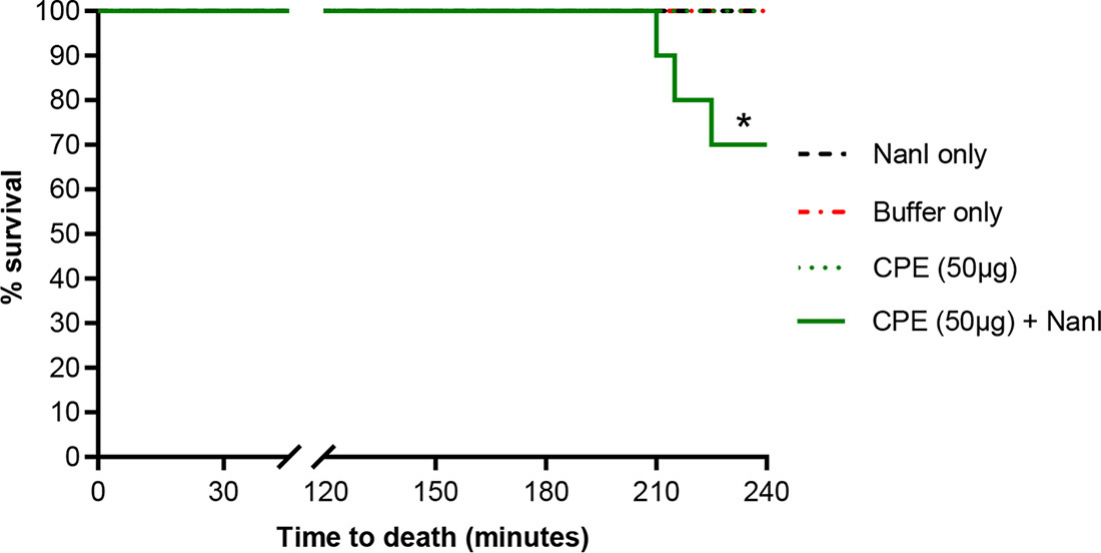
Effects of NanI and CPE in a mouse model of enterotoxemia. Mouse intestinal loops were injected with 1 mL HBSS buffer containing 50 μg CPE plus sialidase (*n* = 10), 50 μg CPE only (*n* = 10), 50 μg sialidase only (*n* = 10), or buffer only (*n* = 10). Intestinal loops were incubated for 4 h or until mice died spontaneously. Time of death was recorded and plotted. Kaplan-Meier survival curves were compared using log-rank analysis. A *P* value of <0.05 was regarded as statistically significant. *, *P* < 0.05 compared with the other three lines.

Results showed that, as expected, no lethality was observed in mice whose intestinal loops were treated for 4 h with either HBSS alone or HBSS containing 50 μg/mL CPE. In addition, no lethality was observed in mice whose intestinal loops were challenged with HBSS containing NanI. However, 30% of mice receiving both 50 μg/mL CPE and NanI showed lethality (Fig. 7), indicating that the copresence of NanI with this CPE dose has a statistically significant synergistic effect on enterotoxemic lethality.

To evaluate if NanI also potentiates the lethality of higher CPE concentrations that (by themselves) are often lethal, the above-described experiments were repeated using 100 μg/mL CPE, which killed about 60% of mice in a previous study (11), using this same enterotoxemia assay. Consistent with results of that previous study, this higher CPE concentration killed ∼40% of the mice in the absence of NanI. While the addition of NanI increased lethality in mice treated with the 100 μg/mL doses of CPE, this effect did not reach statistical significance (Fig. S2).

10.1128/mSphere.00848-21.2 FIG S2 Effects of NanI and CPE in a mouse model of enterotoxemia. Mouse intestinal loops were injected with 1 mL HBSS buffer containing 100 μg CPE plus sialidase (*n* = 10), 100 μg CPE only (*n* = 10), 100 μg sialidase only (*n* = 10), or buffer only (*n* = 10). Intestinal loops were incubated for 4 h or until mice died spontaneously. Time of death was recorded and plotted. Kaplan-Meier survival curves were compared using log-rank analysis. A *P* value of <0.05 was regarded as statistically significant. ns, not significant. Download FIG S2, TIF file, 0.03 MB. Copyright © 2021 Navarro et al. 2021 Navarro et al. https://creativecommons.org/licenses/by/4.0/ This content is distributed under the terms of the Creative Commons Attribution 4.0 International license.

### NanI effects on CPE absorption from the small intestine.

**FIG 8:**
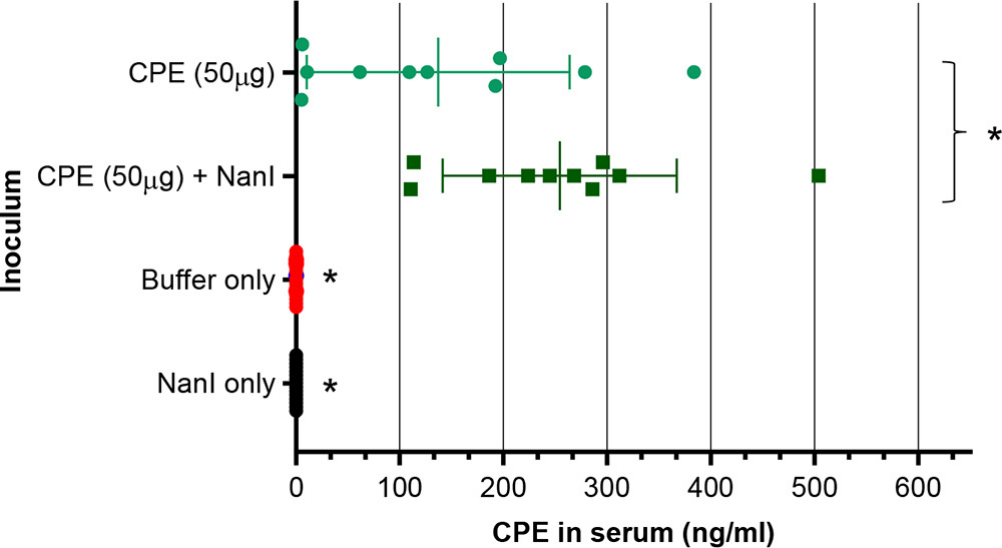
Effects of NanI on CPE absorption from the intestine. Mouse intestinal loops were injected with 1 mL HBSS buffer containing 50 μg of CPE plus NanI (*n* = 10), 50 μg CPE only (*n* = 10), 50 μg NanI only (*n* = 10), or buffer only (*n* = 10). Blood samples were then collected, and serum samples were evaluated for CPE detection and quantitation by enzyme-limited immunosorbent assay (ELISA). Results depict the means of samples from 10 mice per group. Error bars show standard error of the means. *, *P* < 0.05 compared to the other three treatments.

To assess whether the presence of NanI increases lethality of the 50 μg/mL CPE dose by increasing CPE absorption from the intestines, blood was collected from the challenged mice to measure their CPE serum levels (Fig. 8). As expected, no CPE was detected in the serum of mice receiving HBSS with NanI or HBSS alone. Mice receiving a 50 μg/mL dose of CPE in the presence of NanI always had >100 ng/mL CPE in their serum. Importantly, the mean serum concentration of CPE measured in mice challenged with the 50 μg/mL dose of both CPE and NanI was significantly higher than the serum CPE levels present in mice receiving this CPE dose but no NanI.

In comparison, mice treated with HBSS containing both NanI and 100 μg/mL CPE showed more CPE absorption than mice treated with HBSS containing this CPE dose in the absence of NanI, but this effect did not reach statistical significance (Fig. S3).

10.1128/mSphere.00848-21.3 FIG S3 Effects of NanI on CPE absorption from the intestine. Mouse intestinal loops were injected with 1 mL of HBSS buffer containing 100 μg CPE plus sialidase (*n* = 10), 100 μg CPE only (*n* = 10), 100 μg sialidase only (*n* = 10), or buffer only (*n* = 10). Blood samples were then collected, and serum samples were evaluated for CPE detection and quantitation by enzyme-limited immunosorbent assay (ELISA). Results depict the means of samples from 10 mice per group. Error bars show standard errors of the means. ns, not significant (*P* > 0.05) between CPE only versus CPE plus NanI treatments. Download FIG S3, TIF file, 0.04 MB. Copyright © 2021 Navarro et al. 2021 Navarro et al. https://creativecommons.org/licenses/by/4.0/ This content is distributed under the terms of the Creative Commons Attribution 4.0 International license.

### NanI effects on CPE-induced histologic damage to the small intestine.

To test whether the increase in CPE absorption and enterotoxemic death observed for mice cochallenged with both NanI and 50 μg/mL CPE could involve an effect of NanI on CPE-induced intestinal damage, intestinal loops were challenged for 4 h with HBSS, HBSS containing NanI at the NanI activity produced by F4969 in the intestines, HBSS containing 50 μg/mL CPE, or HBSS containing the same NanI activity plus 50 μg/mL CPE. For comparison, the histologic effects on the small intestine of a challenge with 100 μg/mL CPE were also evaluated in the presence or absence of NanI.

**FIG 9:**
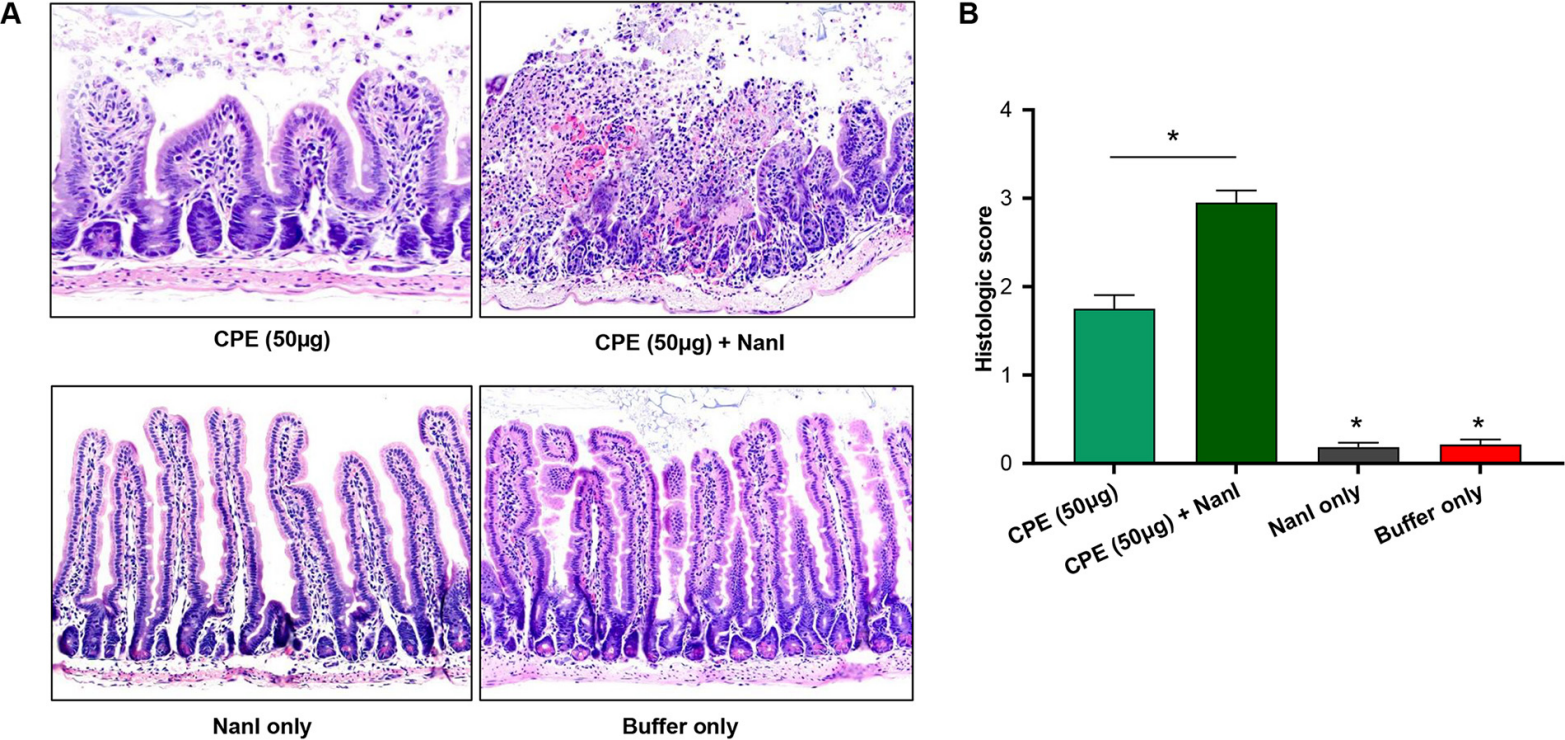
Effects of NanI on CPE-induced intestinal damage. (A) Mouse intestinal loops were injected with 1 mL of HBSS buffer containing 50 μg CPE plus NanI (*n* = 10), 50 μg CPE only (*n* = 10), 50 μg NanI only (*n* = 10), or buffer only (*n* = 10). After 4 h of incubation, or until mice died spontaneously, 4-μm-thick samples of intestinal loops were sectioned and stained with hematoxylin and eosin (H&E). (B) Histological score of intestinal loops injected with the indicated treatment for 4 h. Error bars show standard error of the means. *, *P* < 0.05 compared to the other three treatments.

After those treatments, small intestines from the challenged animals were examined histologically, following the criteria explained in Materials and Methods. As shown in Fig. 9A, no intestinal histologic damage was detected in loops treated with either HBSS or HBSS plus NanI. Treatment with HBSS containing this 50 μg/mL dose of CPE alone caused damage to the mouse small intestinal mucosa. However, this damage was significantly more severe in mice treated with HBSS containing this CPE dose plus NanI, where it was characterized by more severe villus blunting and fusion and epithelial necrosis and desquamation (Fig. 9A and B). No statistically significant differences were detected between intestinal loops treated with HBSS containing 100 μg/mL of CPE with or without the presence of NanI (Fig. S4A and B).

10.1128/mSphere.00848-21.4 FIG S4 Effects of NanI on CPE-induced intestinal damage. (A) Mouse intestinal loops were injected with 1 mL of HBSS buffer containing 100 μg CPE plus sialidase (*n* = 10), 100 μg CPE only (*n* = 10), 100 μg sialidase only (*n* = 10), or buffer only (*n* = 10). After 4 h of incubation, or after mice died spontaneously, 4-μm-thick samples of intestinal loops were sectioned and stained with hematoxylin and eosin (H&E). (B) Histological score of intestinal loops injected with the indicated treatment for 4 h. Error bars show standard errors of the means. ns, not significant (*P* > 0.05) between CPE and CPE plus NanI treatments. Download FIG S4, TIF file, 0.5 MB. Copyright © 2021 Navarro et al. 2021 Navarro et al. https://creativecommons.org/licenses/by/4.0/ This content is distributed under the terms of the Creative Commons Attribution 4.0 International license.

Taken together, the results shown in Fig. 9 indicate that cotreatment with NanI significantly increases intestinal damage caused by the moderate 50 μg/mL CPE dose.

## DISCUSSION

While type F strains are major enteropathogens of humans, their virulence remains incompletely understood beyond appreciating the central importance of CPE (41). The current study reports several findings that offer new insights into type F disease. First, our cell culture results strongly suggest that mucus is a host protective factor against CPE during type F intestinal diseases, which initiates in the mucus-coated intestines. Specifically, our results indicated that MTX-E12 cells, which produce a strong adherent layer of mucus, are significantly less sensitive to CPE than are the HT29 cells from which MTX-E12 cells are derived. The importance of mucus as a protection against CPE was further supported by NAC depletion results.

This study has also identified a counterstrategy that many type F strains can use to overcome the protective effects of mucus. NanI, which is produced by nearly all type F nonfoodborne human GI disease strains and by some type F food poisoning strains, was shown to be active against mucus, i.e., NanI released much more sialic acid from MTX-E12 cells than from HT29 cells. This effect offers an explanation for why the copresence of NanI significantly increased CPE activity proportionately more for MTX-E12 than for HT29 cells. This potentiation involved NanI increasing CPE activity proportionally more for MTX-E12 than for HT29 cells, an effect that likely involves (i) NanI degrading mucus to allow CPE improved physical access to its claudin receptors on cell surfaces and/or (ii) NanI degrading mucus and sialylated host cell surface molecules, which could reduce charge repulsion effects between CPE and the host cell surface, thereby enhancing CPE binding. Similarly, results of this study indicated that NanI degradation of mucus increases permeability for FDs and decreases TEER proportionately more for MTX-E12 cells than for HT29 cells. Also, NanI-mediated enhancement of CPE cytotoxicity was implicated as a major contributor to the decreased TEER and increased permeability of FDs observed in this study.

These *in vitro* cell culture results apparently recapitulate the intestinal situation during CPE treatment based upon the *in vivo* enterotoxemia results of the current study. The copresence of NanI enhanced CPE-induced intestinal damage, which is a consequence of CPE-induced cytotoxicity (42). This NanI enhancement of CPE intestinal damage offers one explanation for the increase in CPE absorption from the intestines that was observed in the presence of NanI. A consequence of this NanI-induced increase in serum CPE levels likely involves the increased lethality noted using a moderate-dose CPE challenge in this study.

This study offers one more important insight into the pathogenesis of type F strains. These bacteria cause disease when they grow and then sporulate in the intestines (1, 5). It is during this *in vivo* sporulation that CPE is produced (1, 5). While CPE production is essential for type F strains to cause pathology in animal models (41), a puzzling observation has been that high levels of purified CPE are needed to cause pathology in animal models. For example, 50 μg/mL purified CPE by itself did not reliably induce lethality in the mouse enterotoxemia assay (see reference 11 and this study), although 100 μg/mL of purified CPE alone is sufficient to induce death in >50% of mice in this assay (reference 11 and this study). Similarly, >50 μg/ml of purified CPE is necessary to reliably cause enteritis in the rabbit small intestinal loop assay (42). These observations have been intriguing because only a few type F strains produce more than 50 μg/mL CPE *in vitro* (43). Similarly, feces from people with type F disease usually contain <50 μg/mL CPE (40).

A possible explanation for this enigma could be that C. perfringens produces accessory factors that can potentiate CPE action. Consistent with that possibility, C. perfringens produces a copious number of extracellular toxins and exoenzymes (2), including proteases, hyaluronidases, and glycosidases such as sialidases that might fit the role of accessory factors to enhance CPE action. As mentioned, type F nonfoodborne GI disease strains, and some type F food poisoning strains, produce NanI as their major sialidase (22). In previous work (29), we showed that NanI is copresent with CPE in sporulating cultures of those NanI-positive type F strains. That previous study also showed that an arbitrary amount of NanI can increase CPE action on Caco-2 cells, which are enterocyte-like cells but make minimal amounts of mucus. However, that previous study did not conduct any *in vivo* experiments to examine whether those cell culture observations have direct pathogenic relevance.

The current study provides the first *in vivo* evidence that C. perfringens accessory factors can potentiate toxins, such as CPE, to enhance pathology. Specifically, this study found that purified NanI can significantly enhance lethal enterotoxemia mediated by purified CPE. This effect was noted using 50 μg/mL CPE, which is a CPE concentration produced by some type F strains *in vitro* and *in vivo* (40, 43). Furthermore, it is possible that other C. perfringens factors present in sporulating cultures may act in combination with NanI during disease to further decrease the amounts of CPE needed to cause pathology. Identifying which accessory factors contribute to type F pathology requires further study and will require the development of a reliable animal infection model allowing type F strain sporulation and CPE action.

## MATERIALS AND METHODS

### C. perfringens isolates, culture media, CPE, NanI, and chemicals.

Type F human nonfoodborne gastrointestinal disease strain F4969 and its *nanI* null mutant were used in the current study; this F4969 *nanI* null mutant was prepared and characterized previously (24). Cooked meat medium (CMM; Difco Laboratories) was used for preparing C. perfringens stocks that were stored at −20°C. The medium used for routine culturing of C. perfringens was fluid thioglycolate (FTG) medium (Difco Laboratories). For experiments, C. perfringens strains were cultured at 37°C under anaerobic conditions.

CPE was purified to nearly 100% homogeneity from type F strain NCTC8238 (ATCC 12916) as described previously (44).

Purified C. perfringens NanI sialidase was purchased from Roche Applied Science. NanI (5 U) was dissolved in 100 μL sialidase reaction buffer (0.05 M Tris-HCl and 1 mM CaCl_2_ [pH 7.2]) and diluted as specified for experiments.

### Cell culture.

Authenticated HT29 cells and MTX-E12 cells were purchased from Sigma-Aldrich. Media used for culturing these cell lines were described previously (34). For use in experiments, these cell lines were grown in 12-mm Costar Transwell plates with 0.4-μm polycarbonate membrane inserts. Media were replaced every 2 days during the 3-week cultivation.

### Measurement of sialidase activity *in vivo* or *in vitro*.

Sialidase activity present in supernatants of intestinal contents from mice challenged with either the wild-type strain F4969 or its *nanI* null mutant was measured based upon modification of a previously described method (23).

Briefly, a 1-mL aliquot of an overnight (∼16 h) FTG culture of wild-type F4969 or the isogenic *nanI* null mutant was centrifuged, and the pellet was then washed in 1 mL HBSS. The resultant suspension (containing ∼10^8^ washed cells) was injected into a ligated mouse small intestinal loop. The experiments involving animals were approved by the University of California, Davis (UC Davis) Committee for Animal Care and Use (permit no. 21729). Approximately equal numbers of male and female (20- to 25-g) BALB/c mice were used. Anesthesia consisted of intraperitoneal administration of 0.2 mL/10 g of body weight of xylazine (0.5 mg/mL) and ketamine (5 mg/mL). Before surgery, iodine solution (Betadine; Purdue Pharma LP) was used to disinfect the abdomen of each animal. An ∼2-cm midline laparotomy was performed before an ∼10-cm-long small intestinal loop was isolated by double ligation of the jejunum; special care was taken to avoid damaging the intestinal blood supply. A new, sterile 1-mL syringe and a 25-gauge needle were used for inoculation into the ligated intestinal loop of each mouse. The abdominal incision was closed in one plane with Super Glue (Henkel Corporation). Mice were divided into two groups receiving 1 mL HBSS containing either the F4969 wild-type or the *nanI* null mutant cells, prepared as described above. A third group of mice received HBSS only. After 10 h of incubation, mice were euthanized, and the intestinal content of each loop was collected.

To measure sialidase activity, an aliquot (20 μL) of supernatant from each intestinal content sample was added to 60 μL of sialidase reaction buffer in a microtiter plate. A 20-μL aliquot of substrate (4 mM 5-bromo-4-chloro-3-indolyl-α-d-*N*-acetylneuraminic acid; Sigma) was added, and the mixture was incubated for 30 min at 37°C. The absorbance at 595 nm (Ab_595_), representing sialidase activity in the supernatant of the intestinal content, was measured using a Bio-Rad microplate reader.

Measurement of sialidase activity in Transwell cultures treated with NanI was performed using the same assay.

### Determination of sialic acid release.

The differences between the detected Ab_595_ (sialidase activity) values measured in small intestinal contents from mice challenged with the wild-type parent versus those challenged with its *nanI* null mutant were determined to be 0.13 (see Results). The equivalent sialidase activity of purified NanI was then applied to HT29 cells or MTX-E12 cells for 1 h at 37°C. After a 1-h treatment with this NanI activity, the sialic acid concentration in culture supernatants was determined using the EnzyChrom neuraminidase assay (Bioassay Systems). Briefly, a 20-μL aliquot of supernatant from the upper chamber of each Transwell chamber was added to 80 μL of working reagent (22.5 L phosphate-buffered saline [PBS] buffer, 55 μL assay buffer, l μL cofactors, l μL enzyme, and 0.5 μL dye reagent, all supplied in the assay kit). Those mixtures were then incubated, with protection from light, for 20 min at 37°C, at which time *A*_590_ was determined. A standard curve of sialic acid concentrations (supplied in the assay kit) was then used to determine the sialic acid concentration generated under each incubation condition.

### Measurement of CPE-induced cytotoxicity in, and CH-1 large complex formation by, HT29 cells and MTX-E12 cells.

In pilot studies, confluent 3-week-old cultures of HT29 and MTX-E12 cell lines in 12-well Transwell plates were treated for 1 h with HBSS with calcium and magnesium but without phenol red (Corning) and containing either 0.5 or 1 μg/mL of purified native CPE.

Later experiments treated HT29 or MTX-E12 cells with HBSS buffer containing 0.5 μg/mL of CPE in the presence or absence of purified NanI that was equivalent to NanI sialidase activity produced by F4969 in the intestines. HBSS alone served as a negative control, and 1% Triton-100 was used as a positive control. Following these treatments, the supernatant was removed from each culture for cytotoxicity detection using the Roche cytotoxicity detection kit (lactate dehydrogenase [LDH]).

To assess CPE large complex (CH-1) formation, HT29 cells or MTX-E12 cells treated as described above were gently collected from the Transwell inserts. Those cells were resuspended in radioimmunoprecipitation assay (RIPA) buffer (Alfa Aesar) containing Benzonase (Millipore Sigma) and proteinase inhibitor (Research Products International [RPI]). Those samples in 5× SDS loading buffer were then used for CPE Western blotting or to show equal protein content.

### CPE Western blot analyses.

To evaluate the presence of CH-1 complex in cells, samples prepared as described above were electrophoresed on 6% polyacrylamide gels containing SDS. Proteins were then electrotransferred onto a polyvinylidene difluoride (PVDF) membrane (Millipore). Those membranes were incubated with CPE anti-rabbit polyclonal antibody to perform CPE Western blotting as described previously (29). After Western blotting, the same PVDF membrane was stained with Coomassie G250 to demonstrate equivalent loading of sample protein content.

### Measurement of transepithelial electrical resistance and monolayer permeability to FD4 or FD40.

To measure transepithelial electrical resistance (TEER), 3-week-old Transwell cultures of HT29 cells or MTX-E12 cells were treated for 1 h at 37°C with HBSS buffer containing 0.5 μg/mL CPE in the presence or absence of purified NanI activity equivalent to NanI sialidase activity produced by F4969 in the intestines. HBSS alone served as a negative control. After those treatments, TEER was measured using the Millicell ERS-2 electrical resistance system (Sigma), and TEER was calculated as described previously (45).

To measure the permeability of FD4 or FD40, cells were washed twice with HBSS buffer. A 0.5-mL aliquot of HBSS buffer or HBSS containing NanI, CPE, or both NanI and CPE, as described above, was added; in addition, the treatment contained 1 mg/mL of either FD4 or FD40 (Sigma-Aldrich) added into the upper chambers of the Transwell plate. Also, 1.5 mL HBSS was added to the lower chamber of each Transwell plate. After 1 h of incubation at 37°C, the concentration of transferred FITC-dextran in the lower chamber was determined using a BioTek Synergy fluorescence multiplate reader (BioTek, Winooski, VT), using excitation at 485 nm and emission at 530 nm. FD4 and FD40 fluxes were calculated as Papp(cmS-1) (45).

### Depletion of extracellular mucus on MTX-E12 cells and Muc5Ac Western blot analysis.

To study the involvement of mucus in the reduced CPE action for MTX-E12 cells, 3-week-old cultures of MTX-E12 cells were treated twice with PBS that did or did not contain 10 mM NAC for 10 min; between each treatment, the cells were incubated with Dulbecco’s modified Eagle’s medium (DMEM) for 1 h.

After this NAC treatment, Muc5Ac Western blot analysis was performed to detect whether the extracellular mucus had been depleted. MTX-E12 cells or MTX-E12 depleted cells were resuspended in phosphate-buffered RIPA lysis buffer (Alfa Aesar) containing protease inhibitor cocktail III (RPI) and Benzonase nuclease (Millipore). After incubation at 4°C for 15 min, the lysates were centrifuged at 20,000 × *g* for 30 min. Supernatants were used for Muc5Ac Western blot analyses, which involved denaturing each supernatant in 5× SDS sample buffer, followed by electrophoresis on an SDS-containing 8% polyacrylamide gel. Samples were then electrotransferred to a PVDF membrane. That membrane was then blocked with 1% Tween-5% bovine serum albumin (BSA)-PBS buffer and incubated overnight with a 1:500 dilution of recombinant anti-mucin 5Ac antibody (EPR16904; Abcam) in 5% BSA-PBS buffer. After washing, the membrane was washed and incubated for 1 h with horseradish peroxidase-conjugated goat anti-rabbit IgG (1: 10,000; Sigma-Aldrich) to detect the Muc5Ac antibody. The substrate used for detecting the bound goat anti-rabbit antibody was Pierce ECL Western blotting substrate. Each PVDF membrane was also stained by Coomassie blue as a loading control.

NAC-treated cells were also tested for their sensitivity to CPE-induced cytotoxicity and CPE effects on TEER, as described earlier.

### Enterotoxemia assay.

Ligated intestinal loops were created in mice to evaluate the effects of CPE in the presence of NanI. A commercially available purified C. perfringens NanI sialidase (Roche) was used at the same NanI sialidase activity level as that detected *in vivo* during F4969 infection (Fig. 1). Briefly, four groups of mice (*n* = 10 per group) received one of the following infections into an intestinal loop: (i) 1 mL HBSS containing CPE (50 μg), (ii) 1 mL HBSS containing CPE (50 μg) plus NanI sialidase, (iii) 1 mL HBSS containing NanI sialidase only, or (iv) 1 mL HBSS buffer only. Death and survival were recorded during a 4-h incubation period. Mice that did not die spontaneously or develop severe clinical signs necessitating earlier euthanasia were euthanized at the end of the 4-h incubation. Mice were kept anesthetized during the whole experiment.

### Histopathology.

After death, samples of intestinal loops were harvested from all mice and fixed by immersion in 10% buffered formalin (pH 7.2) for 24 to 72 h. Sections (4 μm thick) were prepared routinely and stained with hematoxylin and eosin (H&E) before they were microscopically examined by a pathologist, who was not informed of the treatment received by each animal. An overall severity score was assigned to the lesions in each section using an ordinal scale from 0 (no lesions observed) to 4 (most severe). The following parameters were considered to create this score: villus blunting, epithelial desquamation, epithelial cell death, cell death in lamina propria, inflammatory infiltrate, dilation of lymphatic vessels, and submucosal edema.

### Measurement of CPE in serum.

A blood sample was collected via cardiocentesis from all mice while under general anesthesia. Serum was separated and tested for the presence of CPE using a commercial enzyme-limited immunosorbent assay (ELISA) kit (Techlab) according to the manufacturer’s instructions. A 50-μL aliquot of each serum was added to 200 μL of diluent (buffered protein solution plus 0.02% thimerosal). Aliquots (100 μL) of this diluted serum or CPE standards (final CPE range, 0 to 500 ng/mL) were added to wells of a polystyrene assay U-bottomed plate (Falcon) containing 50 μL of conjugate (which was a polyclonal antibody specific for CPE coupled to horseradish peroxidase in a buffered protein solution containing 0.02% thimerosal). Each plate was incubated for 2 h at 37°C and, after five washes, 100 μL of substrate (buffered solution containing tetramethylbenzidine and peroxide) was added to each well. After 15 min of incubation at room temperature, 50 μL of stop solution (0.6 N sulfuric acid) was added to each well. Absorbance was read at 450 nm using a microplate reader (Bio-Rad). Most serum samples were tested twice, with the average of the two readings used for further calculations. Absorbance values for the CPE standards were plotted against known concentrations of CPE. A standard curve was constructed, and the CPE concentration in each serum sample was calculated using the equation of the line of best fit.

### Statistical analyses.

All statistical analyses were performed using R v3.3.1 (for the *in vivo* experiments) and GraphPad 8 (for the *in vitro* experiments). For comparison of treatment, one-way analysis of variance (ANOVA) was applied with *post hoc* analysis using Tukey’s multiple-comparison test for three or more treatments. Student’s *t* test analysis was used for comparisons between two groups. Differences were considered significant when the *P* value was less than 0.05.

## References

[1] McClane BA, Uzal FA, Miyakawa MF, Lyerly D, Wilkins TD. 2006. The enterotoxic clostridia, p 688–752. In Dworkin M, Falkow S, Rosenburg E, Schleifer H, Stackebrandt E (ed), The prokaryotes, 3rd ed. Springer, New York, NY.

[2] Mehdizadeh-Gohari I, Navarro MA, Li J, Shrestha A, Uzal FA, McClane BA. 2021. Pathogenicity and virulence of *Clostridium perfringens*. Virulence 12:723–753. doi:10.1080/21505594.2021.1886777. 33843463 PMC8043184

[3] Uzal FA, Freedman JC, Shrestha A, Theoret JR, Garcia J, Awad MM, Adams V, Moore RJ, Rood JI, McClane BA. 2014. Towards an understanding of the role of *Clostridium perfringens* toxins in human and animal disease. Future Microbiol 9:361–377. doi:10.2217/fmb.13.168. 24762309 PMC4155746

[4] Rood JI, Adams V, Lacey J, Lyras D, McClane BA, Melville SB, Moore RJ, Popoff MR, Sarker MR, Songer JG, Uzal FA, Van Immerseel F. 2018. Expansion of the *Clostridium perfringens* toxin-based typing scheme. Anaerobe 53:5–10. doi:10.1016/j.anaerobe.2018.04.011. 29866424 PMC6195859

[5] McClane BA, Robertson SL, Li J. 2013. *Clostridium perfringens*, p 465–489. In Doyle MP, Buchanan RL (ed), Food microbiology: fundamentals and frontiers, 4th ed. ASM Press, Washington, DC.

[6] Larcombe S, Hutton ML, Lyras D. 2016. Involvement of bacteria other than *Clostridium difficile* in antibiotic-associated diarrhoea. Trends Microbiol 24:463–476. doi:10.1016/j.tim.2016.02.001. 26897710

[7] Carman RJ. 1997. *Clostridium perfringens* in spontaneous and antibiotic associated diarrhoea of man and other animals. Rev Med Microbiol 8:S43–S45.

[8] Centers for Disease Control and Prevention (CDC). 2012. Fatal foodborne *Clostridium perfringens* illness at a state psychiatric hospital—Louisiana, 2010. MMWR Morb Mortal Wkly Rep 61:605–608. 22895383

[9] Bos J, Smithee L, McClane B, Distefano RF, Uzal F, Songer JG, Mallonee S, Crutcher JM. 2005. Fatal necrotizing colitis following a foodborne outbreak of enterotoxigenic *Clostridium perfringens* type A infection. Clin Infect Dis 40:e78-83–e83. doi:10.1086/429829. 15844055

[10] Bamford C, Milligan P, Kaliski S. 2019. Dangers of *Clostridium perfringens* food poisoning in psychiatric patients. S Afr J Psychiatr 25:1339. doi:10.4102/sajpsychiatry.v25i0.1339. 32201630 PMC7081833

[11] Caserta JA, Robertson SL, Saputo J, Shrestha A, McClane BA, Uzal FA. 2011. Development and application of a mouse intestinal loop model to study the *in vivo* action of *Clostridium perfringens* enterotoxin. Infect Immun 79:3020–3027. doi:10.1128/IAI.01342-10. 21628512 PMC3147562

[12] Li J, Paredes-Sabja D, Sarker MR, McClane BA. 2016. *Clostridium perfringens* sporulation and sporulation-associated toxin production. Microbiol Spectr 4. doi:10.1128/microbiolspec.TBS-0022-2015. PMC4920134 27337447

[13] Kitadokoro K, Nishimura K, Kamitani S, Fukui-Miyazaki A, Toshima H, Abe H, Kamata Y, Sugita-Konishi Y, Yamamoto S, Karatani H, Horiguchi Y. 2011. Crystal structure of *Clostridium perfringens* enterotoxin displays features of β-pore-forming toxins. J Biol Chem 286:19549–19555. doi:10.1074/jbc.M111.228478. 21489981 PMC3103334

[14] Briggs DC, Naylor CE, Smedley JG, Lukoyanova N, Robertson S, Moss DS, McClane BA, Basak AK. 2011. Structure of the food-poisoning *Clostridium perfringens* enterotoxin reveals similarity to the aerolysin-like pore-forming toxins. J Mol Biol 413:138–149. doi:10.1016/j.jmb.2011.07.066. 21839091 PMC3235586

[15] Freedman JC, Shrestha A, McClane BA. 2016. *Clostridium perfringens* enterotoxin: action, genetics, and translational applications. Toxins 8:73. doi:10.3390/toxins8030073. PMC4810218 26999202

[16] Robertson SL, Smedley JG, Singh U, Chakrabarti G, Van Itallie CM, Anderson JM, McClane BA. 2007. Compositional and stoichiometric analysis of *Clostridium perfringens* enterotoxin complexes in Caco-2 cells and claudin 4 fibroblast transfectants. Cell Microbiol 9:2734–2755. doi:10.1111/j.1462-5822.2007.00994.x. 17587331

[17] Chakrabarti G, McClane BA. 2005. The importance of calcium influx, calpain and calmodulin for the activation of CaCo-2 cell death pathways by *Clostridium perfringens* enterotoxin. Cell Microbiol 7:129–146. doi:10.1111/j.1462-5822.2004.00442.x. 15617529

[18] Chakrabarti G, Zhou X, McClane BA. 2003. Death pathways activated in CaCo-2 cells by *Clostridium perfringens* enterotoxin. Infect Immun 71:4260–4270. doi:10.1128/IAI.71.8.4260-4270.2003. 12874301 PMC166005

[19] Shrestha A, Mehdizadeh-Gohari I, McClane BA. 2019. RIP1, RIP3, and MLKL contribute to cell death caused by *Clostridium perfringens* enterotoxin. mBio 10:e02985-19. doi:10.1128/mBio.02985-19. 31848291 PMC6918092

[20] Tailford LE, Crost EH, Kavanaugh D, Juge N. 2015. Mucin glycan foraging in the human gut microbiome. Front Genet 6:81. doi:10.3389/fgene.2015.00081. 25852737 PMC4365749

[21] Juge N, Tailford L, Owen CD. 2016. Sialidases from gut bacteria: a mini-review. Biochem Soc Trans 44:166–175. doi:10.1042/BST20150226. 26862202 PMC4747158

[22] Li J, Uzal FA, McClane BA. 2016. *Clostridium perfringens* sialidases: potential contributors to intestinal pathogenesis and therapeutic targets. Toxins 8:341. doi:10.3390/toxins8110341. PMC5127137 27869757

[23] Li J, Sayeed S, Robertson S, Chen J, McClane BA. 2011. Sialidases affect the host cell adherence and epsilon toxin-induced cytotoxicity of *Clostridium perfringens* type D strain CN3718. PLoS Pathog 7:e1002429. doi:10.1371/journal.ppat.1002429. 22174687 PMC3234242

[24] Li J, McClane BA. 2014. Contributions of NanI sialidase to Caco-2 cell adherence by *Clostridium perfringens* type A and C strains causing human intestinal disease. Infect Immun 82:4620–4630. doi:10.1128/IAI.02322-14. 25135687 PMC4249343

[25] Li J, McClane BA. 2014. The sialidases of *Clostridium perfringens* type D strain CN3718 differ in their properties and sensitivities to inhibitors. Appl Environ Microbiol 80:1701–1709. doi:10.1128/AEM.03440-13. 24375134 PMC3957610

[26] Chiarezza M, Lyras D, Pidot SJ, Flores-Díaz M, Awad MM, Kennedy CL, Cordner LM, Phumoonna T, Poon R, Hughes ML, Emmins JJ, Alape-Girón A, Rood JI. 2009. The NanI and NanJ sialidases of *Clostridium perfringens* are not essential for virulence. Infect Immun 77:4421–4428. doi:10.1128/IAI.00548-09. 19651873 PMC2747931

[27] Navarro MA, Li J, McClane BA, Morrell E, Beingesser J, Uzal FA. 2018. NanI sialidase is an important contributor to *Clostridium perfringens* type F strain F4969 intestinal colonization in mice. Infect Immun 86:e00462-18. doi:10.1128/IAI.00462-18. 30297524 PMC6246908

[28] Li J, McClane BA. 2018. NanI sialidase can support the growth and survival of *Clostridium perfringens* strain F4969 in the presence of sialyated host macromolecules (mucin) or Caco-2 Cells. Infect Immun 86:e00547-17. doi:10.1128/IAI.00547-17. 29203541 PMC5778372

[29] Theoret JR, Li J, Navarro MA, Garcia JP, Uzal FA, McClane BA. 2018. Native or proteolytically activated NanI sialidase enhances the binding and cytotoxic activity of *Clostridium perfringens* enterotoxin and beta toxin. Infect Immun 86:e00730-17. doi:10.1128/IAI.00730-17. 29038129 PMC5736825

[30] Nollevaux G, Devillé C, El Moualij B, Zorzi W, Deloyer P, Schneider Y-J, Peulen O, Dandrifosse G. 2006. Development of a serum-free co-culture of human intestinal epithelium cell-lines (Caco-2/HT29-5M21). BMC Cell Biol 7:20. doi:10.1186/1471-2121-7-20. 16670004 PMC1617214

[31] Paone P, Cani PD. 2020. Mucus barrier, mucins and gut microbiota: the expected slimy partners? Gut 69:2232–2243. doi:10.1136/gutjnl-2020-322260. 32917747 PMC7677487

[32] Haines-Menges BL, Whitaker WB, Lubin JB, Boyd EF. 2015. Host sialic acids: a delicacy for the pathogen with discerning taste. Microbiol Spectr 3. doi:10.1128/microbiolspec.MBP-0005-2014. PMC6089508 26350327

[33] Behrens I, Stenberg P, Artursson P, Kissel T. 2001. Transport of lipophilic drug molecules in a new mucus-secreting cell culture model based on HT29-MTX cells. Pharm Res 18:1138–1145. doi:10.1023/A:1010974909998. 11587485

[34] Li J, Navarro MA, Uzal FA, McClane BA. 2021. NanI sialidase contributes to the growth and adherence of *Clostridium perfringens* type F strain F4969 in the presence of adherent mucus. Infect Immun 89:e0025621. doi:10.1128/IAI.00256-21. 34424746 PMC8519267

[35] Smedley JG, Uzal FA, McClane BA. 2007. Identification of a prepore large-complex stage in the mechanism of action of *Clostridium perfringens* enterotoxin. Infect Immun 75:2381–2390. doi:10.1128/IAI.01737-06. 17307943 PMC1865780

[36] Kokai-Kun JF, Benton K, Wieckowski EU, McClane BA. 1999. Identification of a *Clostridium perfringens* enterotoxin region required for large complex formation and cytotoxicity by random mutagenesis. Infect Immun 67:5634–5641. doi:10.1128/IAI.67.11.5634-5641.1999. 10531210 PMC96936

[37] Maares M, Keil C, Koza J, Straubing S, Schwerdtle T, Haase H. 2018. *In vitro* studies on zinc binding and buffering by intestinal mucins. Int J Mol Sci 19:2662. doi:10.3390/ijms19092662. PMC6164875 30205533

[38] Schroeder BO. 2019. Fight them or feed them: how the intestinal mucus layer manages the gut microbiota. Gastroenterol Rep (Oxf) 7:3–12. doi:10.1093/gastro/goy052. 30792861 PMC6375348

[39] Freedman JC, Navarro MA, Morrell E, Beingesser J, Shrestha A, McClane BA, Uzal FA. 2018. Evidence that *Clostridium perfringens* enterotoxin-induced intestinal damage and enterotoxemic death in mice can occur independently of intestinal caspase-3 activation. Infect Immun 86:e00931-17. doi:10.1128/IAI.00931-17. 29685988 PMC6013662

[40] Bartholomew BA, Stringer MF, Watson GN, Gilbert RJ. 1985. Development and application of an enzyme linked immunosorbent assay for *Clostridium perfringens* type A enterotoxin. J Clin Pathol 38:222–228. doi:10.1136/jcp.38.2.222. 2857184 PMC499106

[41] Sarker MR, Carman RJ, McClane BA. 1999. Inactivation of the gene (*cpe*) encoding *Clostridium perfringens* enterotoxin eliminates the ability of two *cpe*-positive *C. perfringens* type A human gastrointestinal disease isolates to affect rabbit ileal loops. Mol Microbiol 33:946–958. doi:10.1046/j.1365-2958.1999.01534.x. 10476029

[42] Smedley JG, Saputo J, Parker JC, Fernandez-Miyakawa ME, Robertson SL, McClane BA, Uzal FA. 2008. Noncytotoxic *Clostridium perfringens* enterotoxin (CPE) variants localize CPE intestinal binding and demonstrate a relationship between CPE-induced cytotoxicity and enterotoxicity. Infect Immun 76:3793–3800. doi:10.1128/IAI.00460-08. 18505809 PMC2493238

[43] Collie RE, Kokai-Kun JF, McClane BA. 1998. Phenotypic characterization of enterotoxigenic *Clostridium perfringens* isolates from non-foodborne human gastrointestinal diseases. Anaerobe 4:69–79. doi:10.1006/anae.1998.0152. 16887625

[44] McDonel JL, McClane BA. 1988. Production, purification, and assay of *Clostridium perfringens* enterotoxin. Methods Enzymol 165:94–103. doi:10.1016/s0076-6879(88)65018-x. 2906731

[45] Mehdizadeh Gohari I, Li J, Uzal FA, McClane BA. 2019. Effects of Claudin-1 on the action of *Clostridium perfringens* enterotoxin in Caco-2 cells. Toxins 11:582. doi:10.3390/toxins11100582. PMC6832201 31601044

[46] Singh U, Van Itallie CM, Mitic LL, Anderson JM, McClane BA. 2000. CaCo-2 cells treated with *Clostridium perfringens* enterotoxin form multiple large complex species, one of which contains the tight junction protein occludin. J Biol Chem 275:18407–18417. doi:10.1074/jbc.M001530200. 10749869

