# Supplementary figures and images for: NanI Sialidase Enhances the Action of Clostridium perfringens Enterotoxin in the Presence of Mucus
Source: mSphere. 2021 Dec 15;6(6):e00848-21. doi: 10.1128/mSphere.00848-21 (PMC8673254; doi:10.1128/mSphere.00848-21)

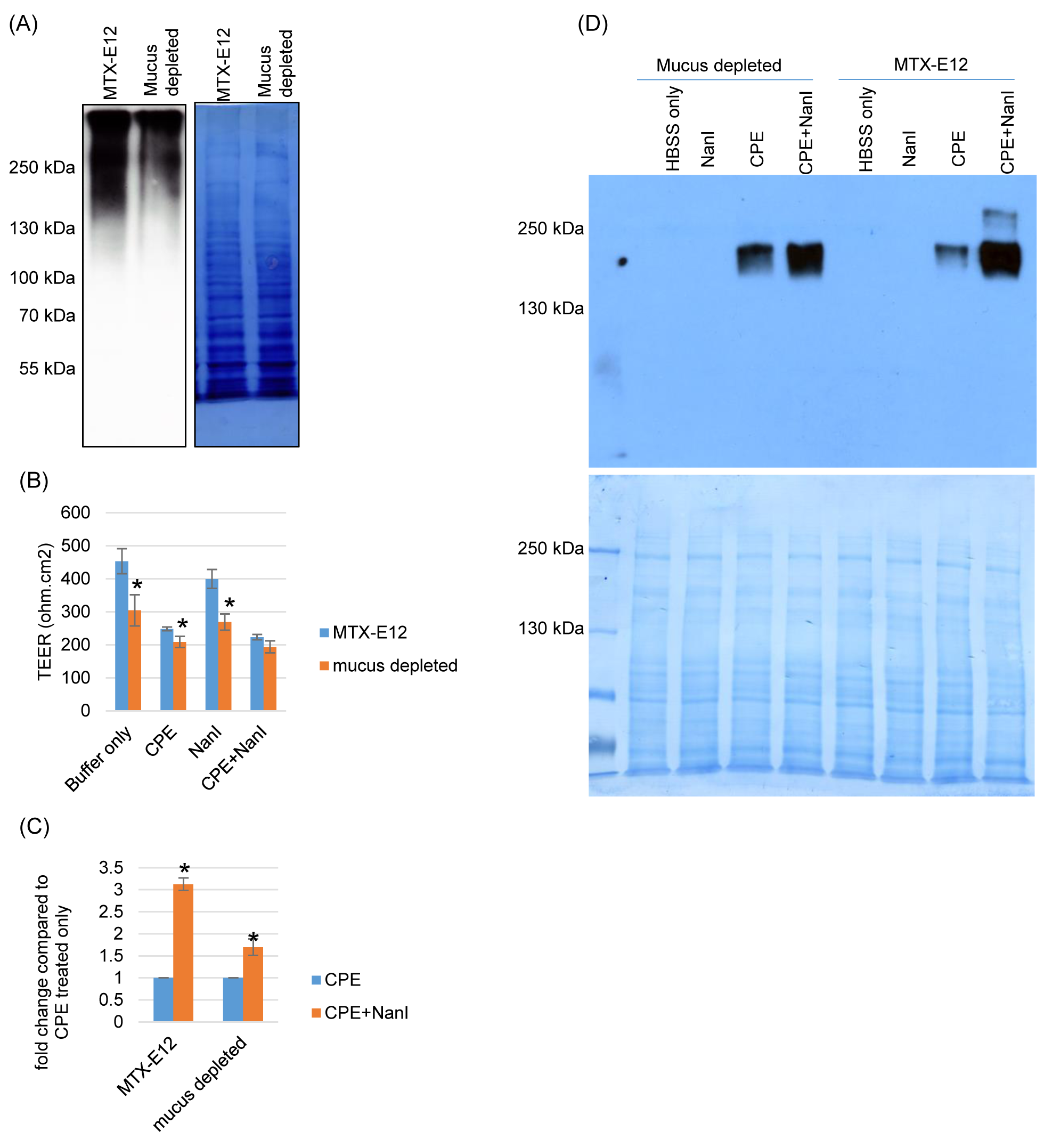

Supplement: FIG S1 [file msphere.00848-21-sf001.tif]

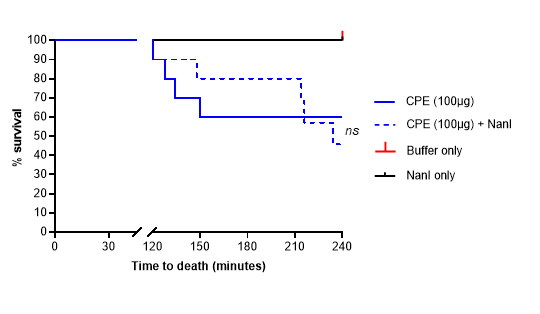

Supplement: FIG S2 [file msphere.00848-21-sf002.tif]

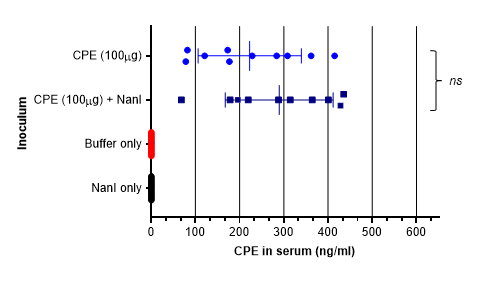

Supplement: FIG S3 [file msphere.00848-21-sf003.tif]

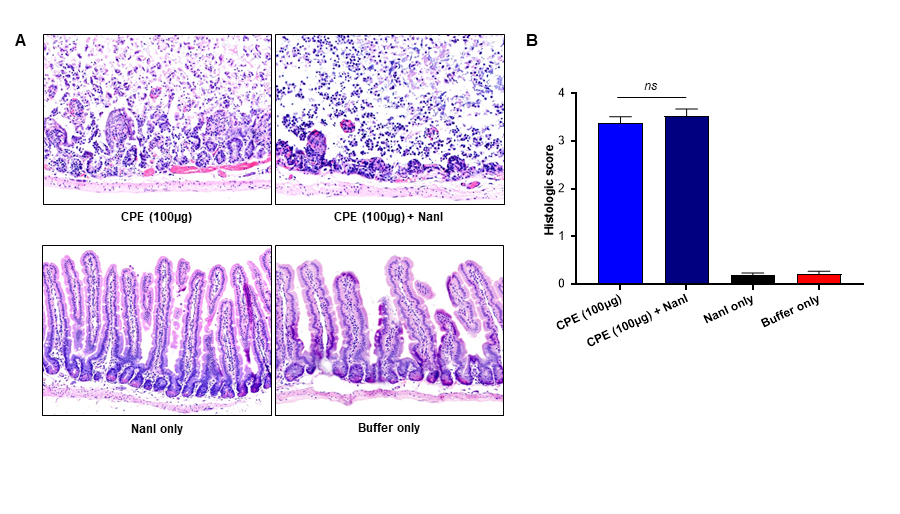

Supplement: FIG S4 [file msphere.00848-21-sf004.tif]
